# Dielectric and Gate Metal Engineering for Threshold Voltage Modulation in Enhancement Mode Monolayer MoS_2_ Field Effect Transistors

**DOI:** 10.1002/adma.202523661

**Published:** 2026-03-17

**Authors:** Lixin Liu, Han Yan, Leyi Loh, Kamal Kumar Paul, Soumya Sarkar, Deepnarayan Biswas, Tien‐Lin Lee, Takashi Taniguchi, Kenji Watanabe, Manish Chhowalla, Yan Wang

**Affiliations:** ^1^ Department of Materials Science & Metallurgy University of Cambridge Cambridge UK; ^2^ Diamond Light Source Harwell Science and Innovation Campus Didcot United Kingdom; ^3^ Research Center for Materials Nanoarchitectonics National Institute for Materials Science Tsukuba Japan; ^4^ Research Center for Electronic and Optical Materials National Institute for Materials Science Tsukuba Japan

**Keywords:** 2D semiconductor, high‐*k* dielectric, interface, threshold voltage

## Abstract

Excellent gate electrostatics in field effect transistors (FETs) based on 2D transition metal dichalcogenide (2D TMD) channels can dramatically decrease static power dissipation. Energy‐efficient FETs operate in enhancement mode with a small and positive threshold voltage (V_th_) for n‐type devices. However, most state‐of‐the‐art FETs based on monolayer MoS_2_ channel operate in depletion mode with negative V_th_ due to doping from the underlying dielectric substrate. In this work, we identify key properties of the semiconductor/dielectric interface (MoS_2_ on industrially relevant high dielectric constant (*k*) HfO_2_, ZrO_2_ and hBN for reference) responsible for realizing enhancement‐mode operation of 2D MoS_2_ channel FETs. We find that hBN and ZrO_2_ dielectric substrates provide low defect interfaces with MoS_2_ that enables effective modulation of the V_th_ using gate metals of different work functions (WFs). We use photoluminescence (PL) and synchrotron X‐ray photoelectron spectroscopy (XPS) measurements to investigate doping levels in monolayer MoS_2_ on different dielectrics with different WF gate metals. We complement the FET and spectroscopic measurements with capacitance‐voltage analysis on dielectrics with varying thicknesses, which confirms that V_th_ modulation in ZrO_2_ devices is correlated with WF of the gate metals – in contrast with HfO_2_ devices that exhibit signatures of V_th_ pinning induced by oxide/interface defect states. Finally, we demonstrate FETs using a 2D MoS_2_ channel and a 6 nm of ZrO_2_ dielectric, achieving a subthreshold swing of 87 mV dec^−1^ and a threshold voltage of 0.1 V. Our results offer insights into the role of dielectric/semiconductor interface in 2D MoS_2_ based FETs for realizing enhancement mode FETs and highlight the potential of ZrO_2_ as a scalable high‐*k* dielectric.

## Graphical abstract

Enhancement‐mode monolayer MoS_2_ FETs with low threshold voltage are essential for low‐power electronics. The threshold voltage can be tuned by the gate metal work function when the semiconductor/dielectric interface is clean. Interfaces between monolayer MoS_2_ and ZrO_2_ or hBN allow effective work‐function modulation of the threshold voltage, in contrast to HfO_2_ where interfacial defects dominate device electrostatics. Spectroscopic and electrical measurements reveal interface‐dependent doping, leading to near‐zero threshold voltage for MoS_2_ FETs with ZrO_2_ dielectrics.

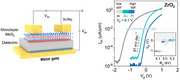

## 1 Introduction

Atomically thin semiconductors, particularly transition metal dichalcogenides (TMDs) such as monolayer MoS_2_, have emerged as promising candidates for next‐generation electronics [1, 2, 3, 4, 5]. While field effect transistors (FETs) based on TMDs hold promise and complementary FETs, 32‐bit microprocessor, gigahertz circuits, and microwave transmitters have been demonstrated [6, 7, 8], most reported n‐type devices operate in depletion mode (threshold voltage, V_th_ < 0), exhibiting considerable channel current (typically > nA/µm) at zero gate bias [8, 9, 10, 11, 12, 13, 14]. FETs that operate in enhancement‐mode (V_th_ > 0) reduce the power consumption by orders of magnitude [15, 16].

Despite the importance of realizing stable and reproducible V_th_ for FETs based on atomically thin TMDs, studies on the underlying mechanisms governing V_th_ remain limited. In silicon metal oxide semiconductor FETs (MOSFETs) beyond the 45 nm node, precise V_th_ is achieved via gate metal WF. That is, gate metals on top of the high *k* dielectric are selected to align near the conduction or valence band edges of silicon to achieve low and symmetric V_th_ values for nMOS and pMOS operation [17, 18]. This strategy has been successfully implemented in FinFETs [19] and gate‐all‐around (GAA) FETs, where matched V_th_ values (≈0.35 V) have been demonstrated [20]. Interface dipole engineering has also been widely studied to develop multiple V_th_ for Si MOSFETs [21, 22]. In emerging technologies such as carbon nanotube FETs, gate WF tuning has achieved V_th_ shifts of ≈0.5 V without introducing dopants [23].

For MoS_2_ FETs, approaches such as solvent doping and the introduction of interface seed layer have been explored to tune the V_th_ of MoS_2_ FETs [9, 24, 25]. While gate WF modulation remains a promising strategy for V_th_ control in FETs based on monolayer MoS_2_, experimental reports often show divergent operation modes when using the same metal gate metals [8, 9, 12, 26, 27, 28, 29]. These discrepancies highlight that the fundamental mechanism governing V_th_ control remains poorly understood. As a result, achieving controlled V_th_ is a key challenge for enabling enhancement‐mode operation in monolayer TMD FETs.

Here, we systematically examine how different dielectrics (hBN, HfO_2_ and ZrO_2_) with different WF gate metals (Al, Au, and Pt) influence the doping of the 2D MoS_2_ semiconducting channel. Photoluminescence (PL) and X‐ray photoelectron spectroscopy (XPS) measurements were used to study the degree of doping and binding energy shift (indicative of Fermi level shift), respectively, in the MoS_2_ channel with different gate metals. We found that metals with different WFs coupled with ZrO_2_ and hBN dielectrics that form a clean semiconductor/dielectric interface can modulate the carrier concentration in the MoS_2_ channel so that the V_th_ can be tuned from negative to positive when going from Al gate metal (WF ≈4.1 eV) to Pt (WF ≈5.2 eV) [30, 31]. In contrast, unintentional electron doping at the interface between HfO_2_ and monolayer MoS_2_ prevents modulation of V_th_, which remains fixed around −0.5 V regardless of gate metal.

## 2 Results and Discussion

**FIGURE 1:**
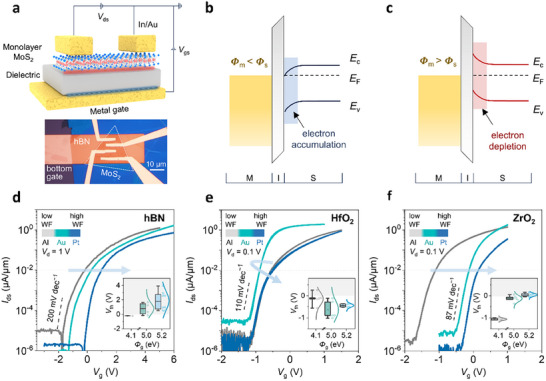
Performance of monolayer MoS_2_ FETs with gate work function engineering. (a) Schematic illustration and optical microscopy (OM) image of the fabricated monolayer MoS_2_ FETs. (b) Energy band alignment of MIS junction with a low WF metal gate. (c) Energy band alignment of MIS junction with a high WF metal gate. (d–f), Transfer characteristics of devices incorporating different gate metals on d, 12 nm of hBN, (e) 6 nm of HfO_2_, and (f) 6 nm of ZrO_2_. The inserted figures show the extracted V_th_ as a function of gate metal work function for multiple devices with different dielectrics.

To investigate the influence of gate metal on devices based on monolayer MoS_2_, we fabricated back‐gated FETs incorporating metal gates with variable WFs. The device configuration is shown in Figure 1a. As illustrated in Figure 1b,c, the band bending in metal‐insulator‐semiconductor (MIS) junctions is modulated by the WF difference between the gate and semiconductor. A low WF metal induces electron accumulation at the semiconductor interface under equilibrium, which leads to a negative shift of V_th_. Conversely, high WF metals deplete electrons and shift V_th_ to positive values. In principle, WF‐induced band bending and carrier redistribution should be even more pronounced in monolayer channels due to their negligible screening thickness. However, atomically thin MoS_2_ is extremely sensitive to its dielectric environment [2, 32], hence the dielectric/semiconductor interface quality critically impacts the effectiveness of gate WF modulation of carriers in the channel. To clarify these effects, we compare FETs with different dielectrics.

The representative transfer characteristics for devices based on monolayer MoS_2_ with 12 nm of hBN, 6 nm of HfO_2_, and 6 nm of ZrO_2_ dielectrics are shown in Figure 1d–f. We use hexagonal boron nitride (hBN) for comparison because it provides an atomically flat, inert interface that preserves the intrinsic properties of MoS_2_ [33, 34]. We observe that as the underlying gate metal is varied from low to high WFs, the transfer curves shift systematically toward positive gate voltages, consistent with the expected modulation of V_th_. The hBN dielectric FETs require relatively large gate voltages (typically > 6 V) to achieve the on state because of the low dielectric constant of hBN, which restricts charge induction efficiency in the channel (details in Figure S1). In contrast, high‐*k* dielectrics such as HfO_2_ and ZrO_2_ greatly enhance electrostatic coupling, enabling relatively low‐voltage operation. The surface morphology and uniformity of the gate stack were systematically examined by AFM, confirming smooth and uniform dielectric thin films (Figure S2). The device characteristics in Figure 1e show that the transfer curves for HfO_2_‐based devices nearly overlap, indicating that the V_th_ is independent of back gate metal. In contrast, V_th_ shifts toward positive values with increasing gate metal work function with ZrO_2_ dielectric, following the same trend as hBN FETs but operating at lower biases. Detailed statistical analyses of V_th_ for multiple devices with different gate metals are shown in the inserted figures (Figure 1d–f; Figure S3). V_th_ exhibits a linear dependence with metal gate work function when hBN is used as the dielectric. However, hBN devices show large variations from device to device because the viscoelastic transfer of mechanically exfoliated hBN for device fabrication introduces air gaps and surface residue. ZrO_2_‐based FETs also exhibit a monotonic increase in V_th_ with increasing gate WF, showing approximately a 1.0 V shift in V_th_ using low and high WF metals. Pt‐gated ZrO_2_ devices operate in enhancement mode with an average V_th_ of ≈0.1 V. FETs using ZrO_2_ as the dielectric were reproducible in their performance and trend. In contrast, HfO_2_‐based devices exhibit a nearly fixed, negative V_th_ of approximately −0.5 V, independent of the gate metal work function. Furthermore, as indicated in Figure 1d–f and Figures S4 and S5, ZrO_2_‐based FETs exhibit the lowest subthreshold swing (SS) values. The favorable electrical performance such as sub 1 V operating voltage, enhancement‐mode operation (average V_th_ ≈0.1 V), and low SS (≈87 mV dec^−1^), demonstrates that ZrO_2_‐based MoS_2_ FETs with Pt gates closely align with the performance requirements for state‐of‐the‐art low‐power FETs [3].

**FIGURE 2:**
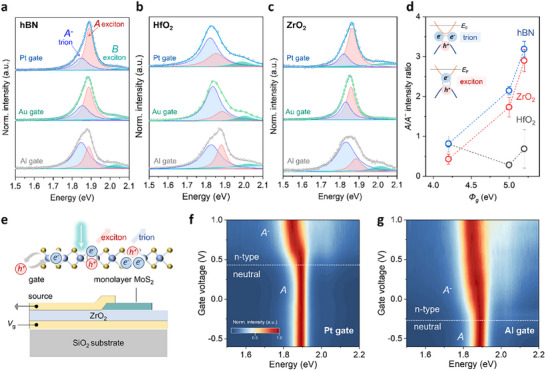
PL analysis of monolayer MoS_2_ with dielectric and gate metal engineering. (a–c) PL spectra of monolayer MoS_2_ on gate metals with different WFs, measured on a, hBN, b, HfO_2_, and (c) ZrO_2_ dielectrics. Changes in exciton and trion emission indicate modulation of carrier density by gate WF. (d) Extracted exciton‐to‐trion intensity ratio as a function of gate WF, extracted from multiple measurement points across different dielectrics. Inset: Schematic illustration of exciton and trion binding states, where trions form through Coulomb binding of an exciton with an additional electron. (e) Schematic of the gate‐dependent PL device used for dynamic carrier modulation studies. (f,g) 2D color maps of gate‐dependent PL for MoS_2_ on ZrO_2_ dielectric with f, low‐work‐function Al gate and g, high‐work‐function Pt gate.

Photoluminescence (PL) spectroscopy measurements of monolayer MoS_2_ were carried out to assess doping levels induced by different gate metals by comparing the relative intensities of neutral exciton (A) and negatively charged trion (A*^−^*) peaks [35, 36]. The PL spectra for MoS_2_ with hBN dielectric and different metal gates are plotted in Figure 2a and Figure S6. A clear transition is observed from trion‐dominant emission with the Al gate to exciton‐dominant peak with the Pt gate. For MoS_2_ on HfO_2_ (Figure 2b), the PL spectra remain trion‐dominant across all gate metals, showing negligible variation with gate WFs. These results indicate that interfacial interactions between HfO_2_ and monolayer MoS_2_ lead to substantial electron doping, rendering the channel resistant to depletion – even with a high work function gate. In contrast, we observed a pronounced tunability in the exciton‐to‐trion ratio, similar to that on hBN, with high‐*k* ZrO_2_ as the dielectric (Figure 2c). Figure 2d presents the exciton‐to‐trion ratio, an optical indicator of electron density in monolayer MoS_2_, as a function of gate work function for the three different dielectric substrates. Effective WF‐induced carrier modulation is evident only in the hBN and ZrO_2_ systems, consistent with the device characteristics.

Gate‐dependent PL measurements were conducted to further validate the influence of electrostatic modulation on exciton/trion dynamics. The device configuration used for these measurements is shown in Figure 2e. The gate tunability of the PL with ZrO_2_ dielectric and Pt and Al gates is illustrated by Figure 2f,g, where the PL emission evolves from trion‐dominant (n‐type) to exciton‐dominant (neutral) as the gate‐induced electron density decreases. The voltage at which this transition occurs is positive for Pt‐gated devices and negative for Al‐gated devices, indicating that the MoS_2_ channel is near charge neutrality under zero bias for the Pt gate, but remains electron‐doped for the Al gate. The voltage difference at which the transition occurs between Pt gate and Al gate devices is ≈0.8 V (details in Figures S7 and S8). A similar gate‐dependent trend is also observed in devices using hBN as the dielectric, as presented in Figures S9 and S10. To further demonstrate gate work function engineering for V_th_ modulation with a high‐quality MoS_2_ /ZrO_2_ interface, an additional gate metal (Ag) was investigated. As summarized in Figure S11, the transfer characteristics and corresponding PL spectra collected from multiple devices exhibit a systematic linear correlation among V_th_, excition‐to‐trion ratio, and gate metal work function.

**FIGURE 3:**
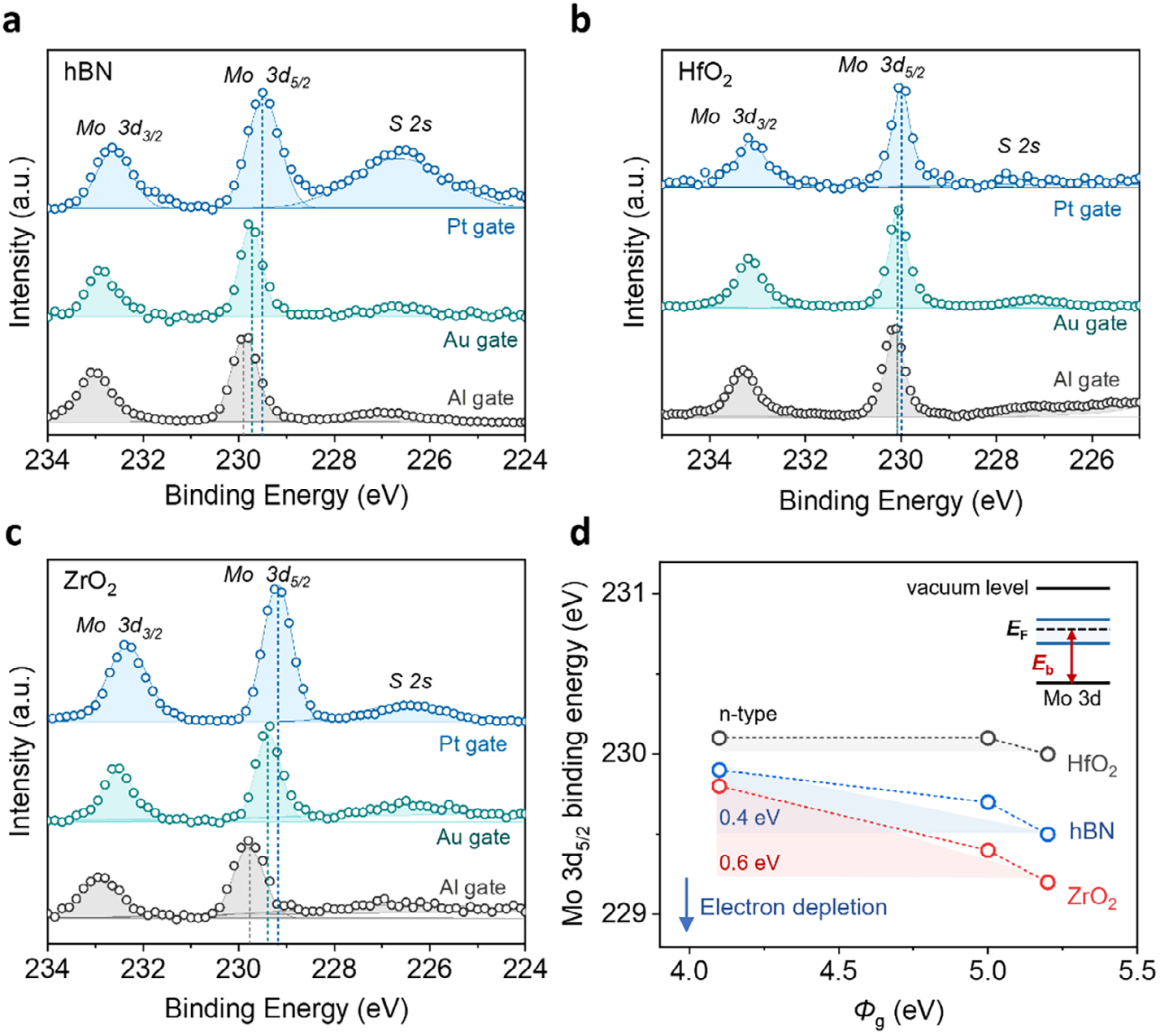
Synchrotron XPS analysis of Fermi level modulation in monolayer MoS_2_. (a–c) High‐resolution XPS spectra of monolayer MoS_2_ measured with different back gate metals buried under a, hBN, b, HfO_2_, and c, ZrO_2_ dielectrics. (d) Extracted binding energy of the Mo 3*d*_5/2_ peak as a function of gate work function. The Mo 3*d*_5/2_ binding energy shifts indicate the relative position of the Fermi level with respect to the conduction band.

To directly quantify the Fermi level modulation of monolayer MoS_2_ induced by different metal gates, we performed synchrotron X‐ray Photoelectron Spectroscopy (XPS) on devices with the three dielectric substrates. Specifically, we measured the binding energy of the Mo 3*d*, which reflects the energy difference between the Fermi level and the Mo 3*d* core level (as illustrated in the inset of Figure 3d) [37]. As the work function of the metal gate increases, a systematic drop in the Mo 3*d* binding energy is observed across all three dielectrics, indicating the absence of electron doping in monolayer MoS_2_ (Figure 3a–c). However, the extent of this Fermi level modulation varies depending on the dielectrics, as summarized in Figure 3d. All spectra were calibrated to C 1*s* (Figure S12). It is evident that ZrO_2_ enables the most effective gate control, where an increase in gate metal work function by ≈1.1 eV leads to a downward Fermi level shift of ≈0.6 eV in MoS_2_. In contrast, the modulation achieved on HfO_2_ is significantly weaker. With identical gate metal configurations, MoS_2_ on HfO_2_ consistently exhibits higher binding energy compared to those on ZrO_2_ and hBN, indicating a pronounced electron‐doping effect. Notably, even with a high work function Pt gate, the Fermi level position of MoS_2_ on HfO_2_ remains relatively high compared to Al‐gated MoS_2_ on hBN or ZrO_2_, suggesting that effective electron depletion is challenging on HfO_2_, which is consistent with the observations in PL measurements and FET characteristics. The energy difference between the Fermi level and the valence band maximum extracted by linear extrapolation of the valence band edge spectra is illustrated in Figure S13. Based on this energy difference, the carrier concentration is estimated using the formula:$n=N_{c}\;\ln\left[1+\exp(\frac{E_{F-}E_{i}}{kT})\right]$ (see supplementary information for details) [38, 39]. The analysis reveals tunability of up to ≈10^13^ cm^−2^ carrier concentration with gate metal in ZrO_2_‐based devices and up to ≈10^12^ cm^−2^ in devices using hBN dielectrics. These values are consistent with the electrical measurements and gate‐dependent PL result summarized in Table S1.

**FIGURE 4:**
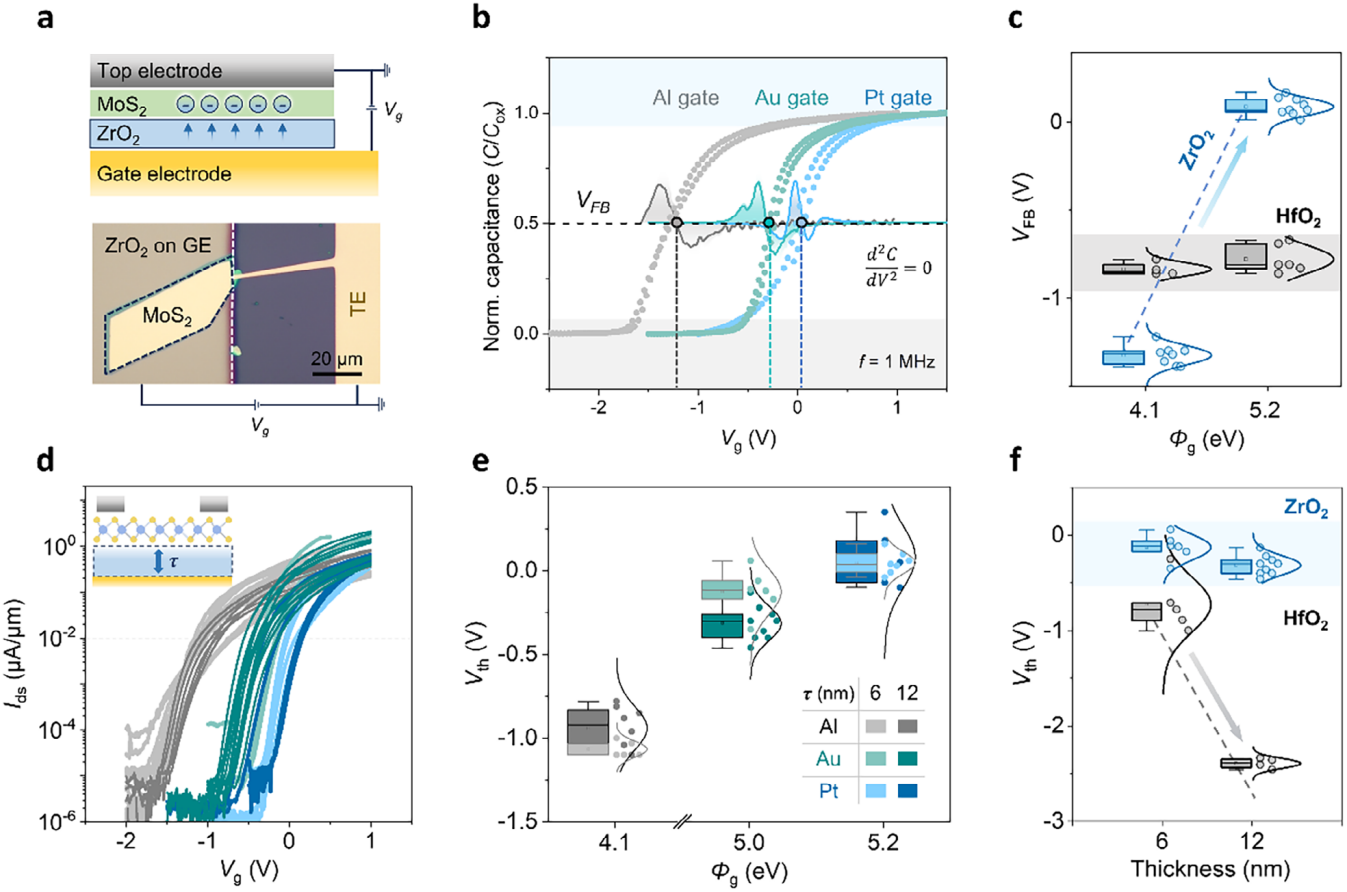
Flat‐band voltage extraction and dielectric‐thickness‐dependent V_th_ modulation in ZrO_2_ and HfO_2_ devices. (a) Schematic and OM image of a representative MOSCAP device. (b) Normalized high‐frequency C‐V measurements for ZrO_2_‐based MOSCAPs with varying bottom metal gates, along with the corresponding second‐derivative curves used to extract V_FB_ at the infection point. (c) Extracted V_FB_ values from multiple devices with Al and Pt gate metals on ZrO_2_ and HfO_2_. (d) Transfer curves with different ZrO_2_ thicknesses (6 nm, lighter curves; 12 nm, darker curves). (e) Extracted V_th_ for the corresponding devices in (d). (f) V_th_ as a function of dielectric thickness for ZrO_2_ and HfO_2_, using Au‐gated devices as an example.

We further investigated key factors in V_th_ modulation for transistors based on monolayer MoS_2_. In MOSFETs, the V_th_ can be expressed as:

$$\;V_{th}=2\Phi_{F}+\gamma \sqrt{2\Phi_{F}}+V_{FB}$$

where Φ_*F*_ is the Fermi potential, γ is referred to as the body‐effect coefficient and V_FB_ is the flat band voltage. To estimate V_FB_, we fabricated metal‐oxide‐semiconductor capacitors [40] (MOSCAP) using mechanically exfoliated few‐layer MoS_2_ – the device structure is shown in Figure 4a. High‐frequency capacitance‐voltage (C‐V) characteristics (Figure 4b) reveal the expected transition from depletion to accumulation. In the depletion regime, the effective capacitance is dominated by the series combination of MoS_2_ and ZrO_2_, whereas in the accumulation regime, the ZrO_2_ capacitance becomes dominant. Detailed analysis of capacitance evolution is provided in Figure S14. Importantly, a clear positive shift in the C‐V curves is observed when varying the gate metals. The extracted V_FB_, determined from the inflection points [41], changes from −1.2 to 0.1 V, which is in good agreement with the V_th_ extracted from FET measurements. This consistency confirms that ZrO_2_ enables effective gate work function modulation with minimal interface or oxide state trapping. In contrast, MOSCAPs with HfO_2_ exhibit fixed V_FB_ values around−0.8 V, independent of the gate metal (Figure 4c; Figures S15 and S16). More data points are provided in Figure S17 to confirm the reproducibility.

Since V_FB_ Arises from both the Work Function Difference and the Interface/Oxide Charge, according to where Φ_*g*_ − Φ_*s*_ is the work function difference between metal gate and the semiconductor channel, *Q_ox_* is oxide charge density, and *C_ox_* is the oxide capacitance. We further fabricated FETs with varying dielectric thicknesses to decouple the effect of Q_ox_. For ZrO_2_‐based devices, the transfer curves and extracted V_th_ remain nearly independent of oxide thickness, demonstrating that Q_ox_ is negligible and that the V_th_ modulation arises almost mainly from the gate‐WF difference (Figure 4d,e). In contrast, HfO_2_‐based devices demonstrated a negative shift in V_th_ with dielectric thickness, highlighting significant contribution from interface trapped charges (Figure 4f; Figures S18 and S19). Together, these results demonstrate that ZrO_2_ enables reliable gate‐work‐function‐driven V_th_ engineering, whereas HfO_2_ exhibits interface‐state‐dominated behavior that suppresses effective V_th_ modulation.

## 3 Conclusion

In summary, we demonstrate modulation of threshold voltage in monolayer MoS_2_ FETs using gate metals with different work functions. Our results highlight that this approach is only effective if the dielectric/semiconductor interface is mostly free of defects. Among the three dielectrics, ZrO_2_ was found to provide a clean and well‐coupled interface that allows tuning of carrier concentration in monolayer MoS_2_ with gate metal work function variation. As a result, ZrO_2_‐based FETs exhibit linear V_th_ modulation with gate metal work function, near‐ideal subthreshold swing, and enhancement‐mode operation when paired with high‐work‐function gate metals such as Pt. These findings provide design guidelines for monolayer MoS_2_‐based FETs that operate in enhancement mode.

## 4 Experimental Section

### 4.1 Sample Preparation

Monolayer MoS_2_ was synthesized through chemical vapor deposition (CVD) using MoO_3_ and sulfur as precursors. Specifically, 60 mg of sulfur powder was placed in an alumina boat located at the upstream edge of the furnace. A separate alumina boat containing 5 mg of MoO_3_ was positioned at the center of the tube. Prior to growth, SiO_2_/Si substrates were spin‐coated with 0.5 mg/mL NaOH solution, serving as the growth promoter. The growth temperature was initially raised from room temperature to 120°C over 3 min and held for 20 min to purge residual gases. Subsequently, the system was ramped to the growth temperature of 720°C, while the MoS_2_ growth was carried out for 12 min. After growth, the sulfur source was promptly removed from the hot zone, and the furnace lid was opened to allow rapid cooling in ambient air. During the temperature ramp‐up and cooldown (T < 650°C), a nitrogen flow of 460 sccm was maintained to ensure an inert atmosphere, while during the high temperature growth phase (T ≥ 650°C), the flow rate was reduced to 60 sccm to optimize reaction kinetics.

### 4.2 Dielectrics

hBN flakes were mechanically exfoliated from bulk single crystals (NIMS, Japan). To minimize surface residue, exfoliation was performed using low‐adhesion Ultron blue tape followed by transfer with a home‐made PDMS stamp. The thicknesses of selected flakes were measured by AFM and found to be approximately 12 nm. ZrO_2_ films were deposited using E‐beam evaporation (Nexdep, Angstrom Engineering). The chamber base pressure was maintained at 5 × 10^−6^ Torr, and the deposition rate was controlled at 0.1 Å/s to ensure uniform film quality. Characterizations of the ZrO_2_ dielectrics are given in Figure S16. HfO_2_ films were deposited through atomic layer deposition (Fiji G2, Veeco). The whole process includes 60 cycles, yielding a film thickness of 6 nm.

### 4.3 Device Fabrications

Bottom gate electrodes (Al, Au, and Pt) were patterned by standard photolithography, followed by the deposition of a 3 nm Ti adhesion layer and 12 nm of metal film using E‐beam evaporation. After gate patterning, the dielectric layers were either transferred (for hBN) or deposited (for ZrO_2_ and HfO_2_) on top of the gate electrodes. Monolayer MoS_2_ flakes were picked up and transferred by the PDMS stamp method [42]. Following the transfer, the samples were spin‐coated with a double‐layer resist of MMA/PMMA. Source and drain electrodes were patterned with standard electron‐beam lithography (EBL), followed by deposition of In/Au (6/60 nm) to avoid damage on monolayer channels and ensure low‐contact resistance.

### 4.4 Measurements

PL spectra were acquired using a confocal Raman/PL system (LabRAM Odyssey, Horiba) under the ×100 objective lens with the incident laser of 532 nm at 52 µW of incident power, which is sufficiently low to avoid photothermal damage to the monolayer samples. Gate‐dependent PL measurements were performed by applying a voltage bias using a source meter (Keithley 2450). During these measurements, the PL signals were collected using the ×50 objective lens, and the excitation power was set to 0.45 mW. All measurements were conducted under ambient conditions. The surface morphology was characterized by atomic force microscopy (AFM; Bruker Dimension Icon) operated in PeakForce tapping mode using ScanAsyst‐Air probes.

Synchrotron X‐ray photoelectron spectroscopy measurements were conducted at the Diamond Light Source (UK), Beamline I09. The incident X‐ray beam spot size was approximately 15 µm × 35 µm. Photon energies ranged from soft X‐ray (1000 eV) to hard X‐ray (3000 eV). All binding energies were calibrated against the standard C 1*s* peak (284.8 eV) to ensure consistency. The samples consisted of CVD‐grown monolayer MoS_2_ transferred onto various dielectric/gate stacks. To eliminate charging effects and ensure accurate energy referencing, all flakes were electrically grounded to the conductive sample holders during measurements.

Electrical measurements were performed with a Keithley 4200 semiconductor parameter analyzer. Prior to measurements, the chamber was pumped down to 10^−6^ Torr and maintained under vacuum for over 1 h to minimize the influence of surface adsorbates and ensure measurement stability.

### 4.5 FET Performance Calculation

The V_th_ was extracted at the drain current of 10 nA/µm from transfer curves.

The SS was extracted from the slope of logarithmic transfer characteristics:

$$SS=\frac{dV_{g}}{dlogI_{d}}$$

## Author Contributions

Y.W. and M.C. conceived the project. Lixin Liu and Y.W. designed and performed the majority of experiments. H.Y. prepared the ALD samples and assisted with analyze the electrical characterization. Leyi Loh contributed to PL measurements and analysis. S.S. and K.K.P. assisted with gate‐dependent PL experiments. Lixin Liu, H.Y., Y.W., and D.P. carried out the synchrotron XPS measurements with support from T.L. Lixin Liu worked on the figures with input from all co‐authors. Lixin Liu, Y.W., and M.C. wrote the manuscript with contributions and feedback from all authors.

## Conflicts of Interest

The authors declare no conflict of interest.

## Supporting information




**Supporting File**: adma72827‐sup‐0001‐SuppMat.docx.

## Data Availability

The data that support the findings of this study are available in the supplementary material of this article.
