# Supplementary material for: Dielectric and Gate Metal Engineering for Threshold Voltage Modulation in Enhancement Mode Monolayer MoS2 Field Effect Transistors
Source: Adv Mater. 2026 Mar 17;38(21):e23661. doi: 10.1002/adma.202523661 (PMC13073085; doi:10.1002/adma.202523661)
Supplement: Supplementary file 1 — Supporting File: adma72827‐sup‐0001‐SuppMat.docx. [file ADMA-38-e23661-s001.docx]

Supplementary information

**Dielectric and Gate Metal Engineering for Threshold Voltage Modulation in Enhancement Mode Monolayer MoS_2_ Field Effect Transistors**


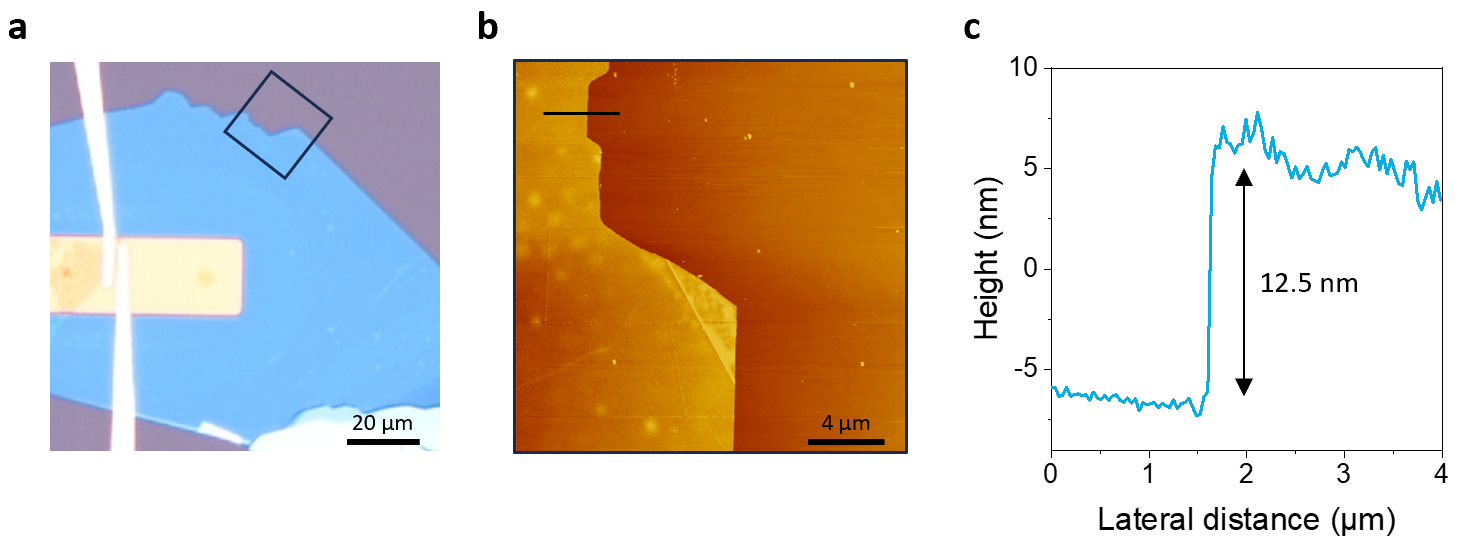


**Figure S1** Thickness characterization of the hBN dielectric. (a) OM image of a representative back-gated MoS_2_ FET using hBN as the dielectric. (b) Atomic force microscopy (AFM) image of the area marked by the black box in (a). (c) Height profile extracted along the line in (b), showing a measured hBN thickness of approximately 12.5 nm. The EOT is calculated using the relation: EOT= 𝑡_hBN_×𝜀_SiO2_/𝜀_hBN_. This yields an EOT of approximately 10 nm.


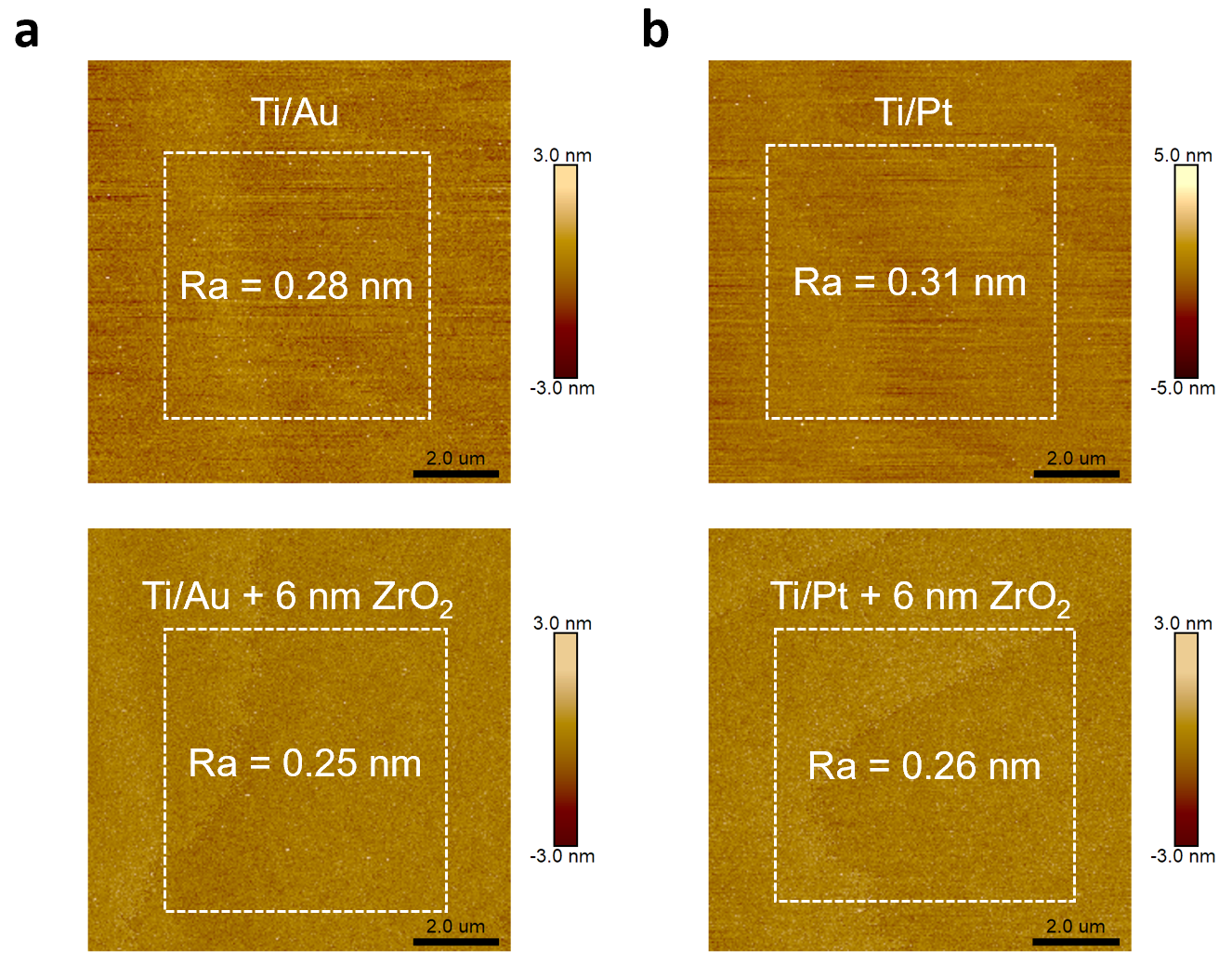


**Figure S2** Surface quality of ZrO_2_ film. a, AFM images of Ti/Au electrodes before and after deposition of ZrO_2_. b, AFM images of Ti/Pt electrodes before and after deposition of ZrO_2_. The low surface roughness in both cases indicates that the ZrO_2_ films are uniform and of high quality.


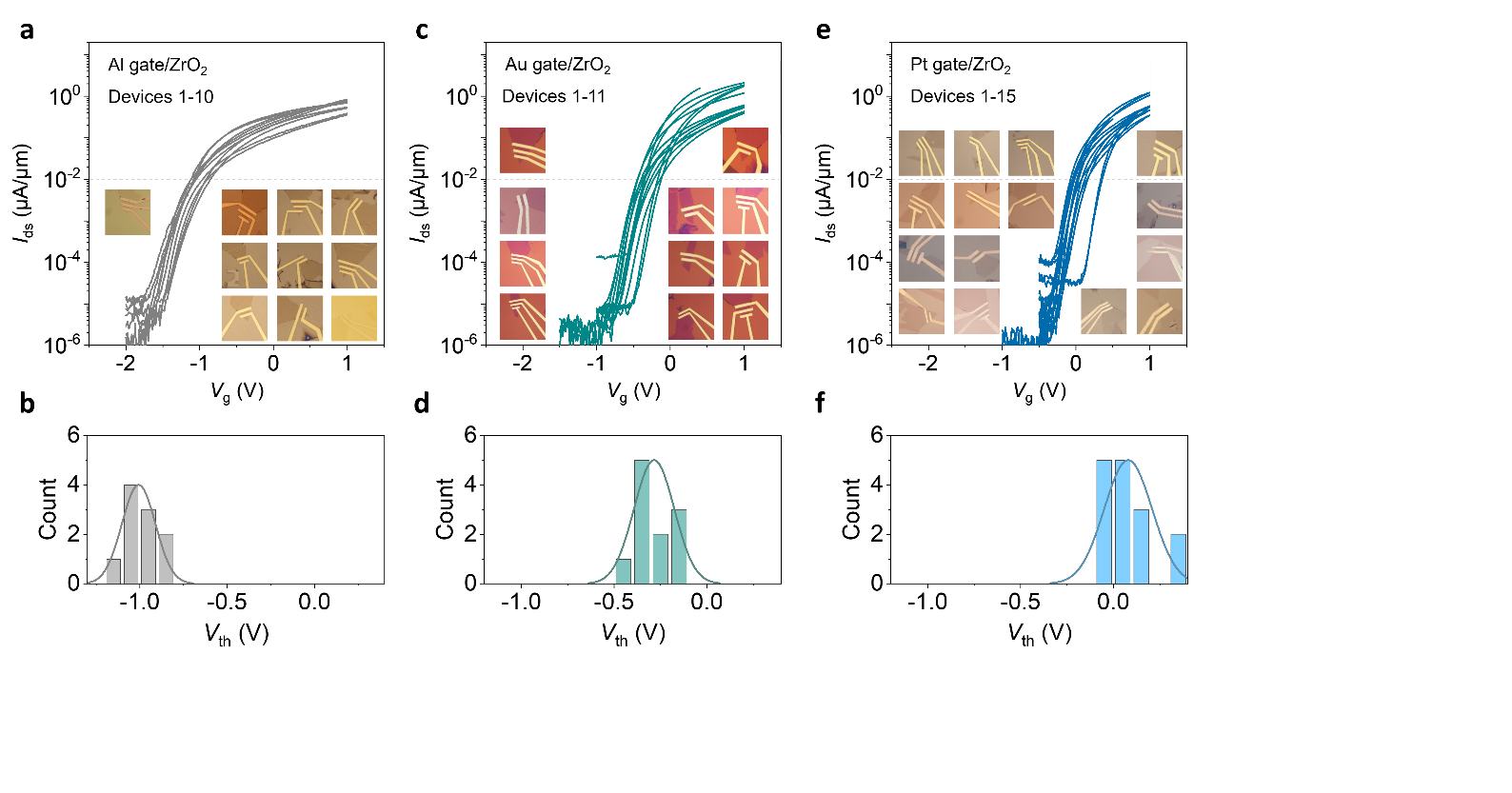


**Figure S3** Sample-to-sample variation in electrical performance of monolayer MoS_2_ FETs. Transfer characteristics and extracted V_th_ distribution of representative devices with an a-b, Al gate. c-d, Au gate. e-f, Pt gate. Insets show the corresponding OM images of the measured devices.


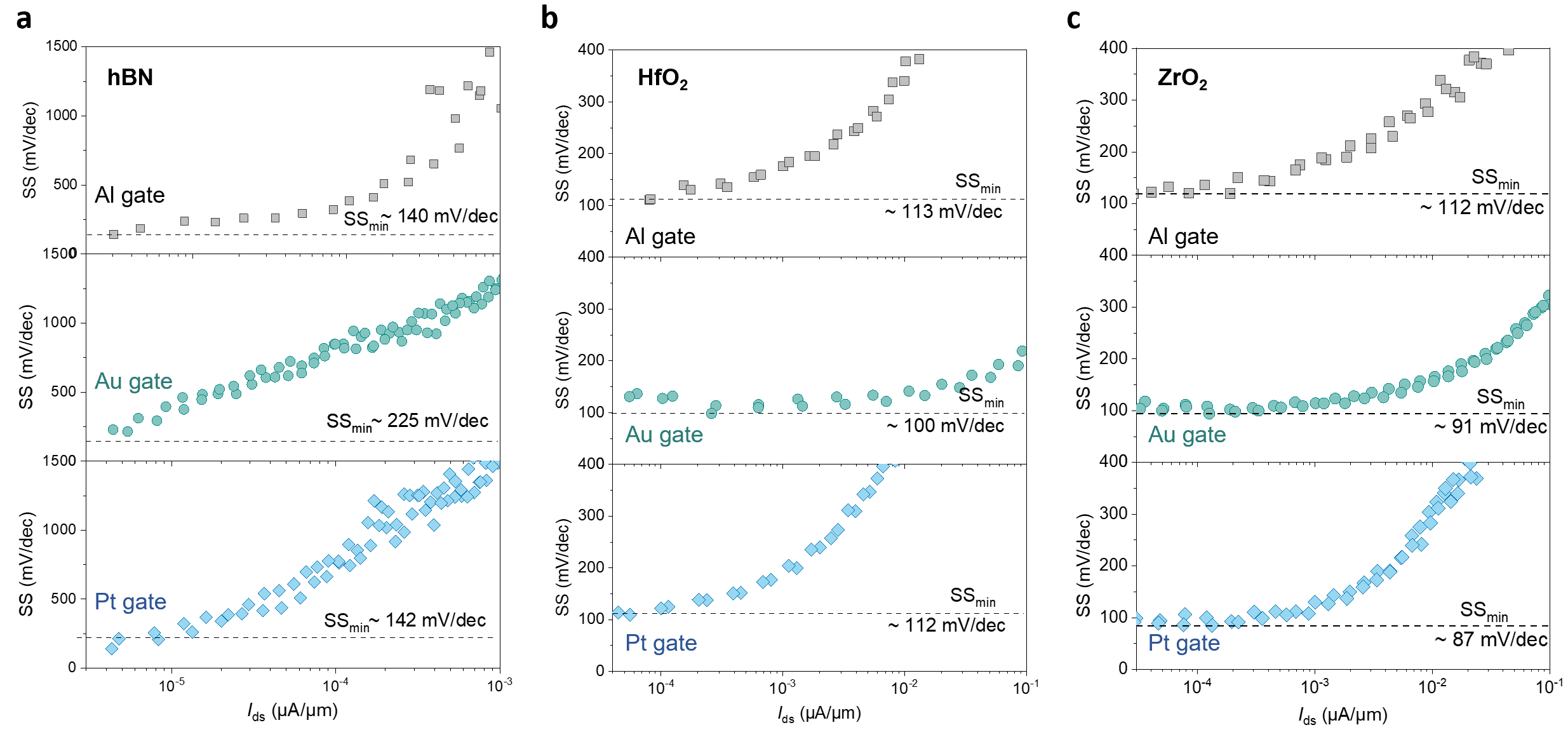


**Figure S4** Extracted SS values for devices fabricated on (a) hBN, (b) HfO_2_, and (c) ZrO_2_ dielectrics with varying gate metals, derived from the transfer characteristics shown in **Figure. 1d–f**.


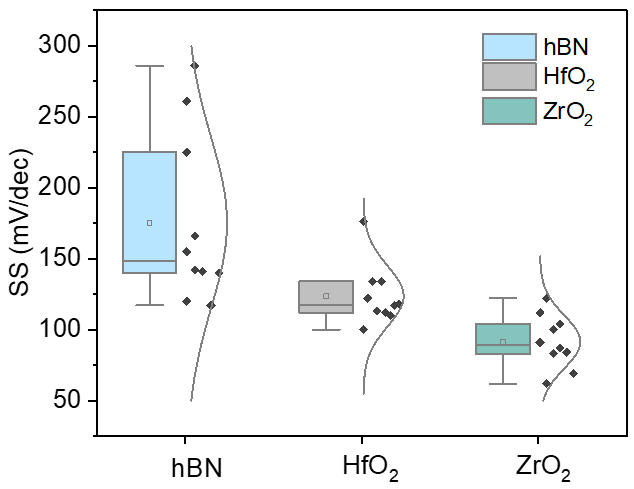


**Figure S5** Statistical analysis of SS values extracted from multiple monolayer MoS₂ devices fabricated on hBN, HfO_2_, and ZrO_2_. ZrO_2_-based devices exhibit the lowest average SS, highlighting efficient gate control and low interfacial scattering.


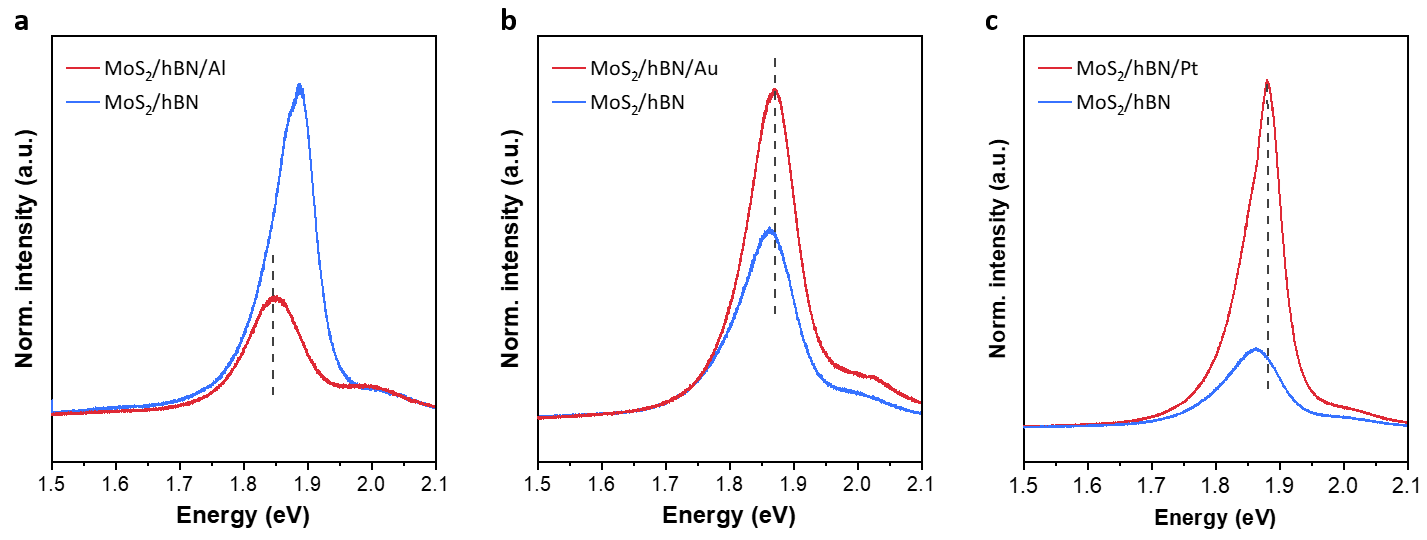


**Figure S6** PL spectra of monolayer MoS_2_ on hBN with (a) Al, (b) Au, and (c) Pt gates beneath, compared to the reference without a gate. In the absence of a gate (pure hBN), MoS_2_ exhibits a relatively neutral state. The introduction of a low-work-function Al gate induces significant electron accumulation, leading to a trion-dominated emission characterized by a redshifted peak and reduced intensity. Conversely, high-work-function gates such as Au and Pt suppress excess electron injection, resulting in exciton-dominated PL with blueshifted peaks and enhanced emission intensity.


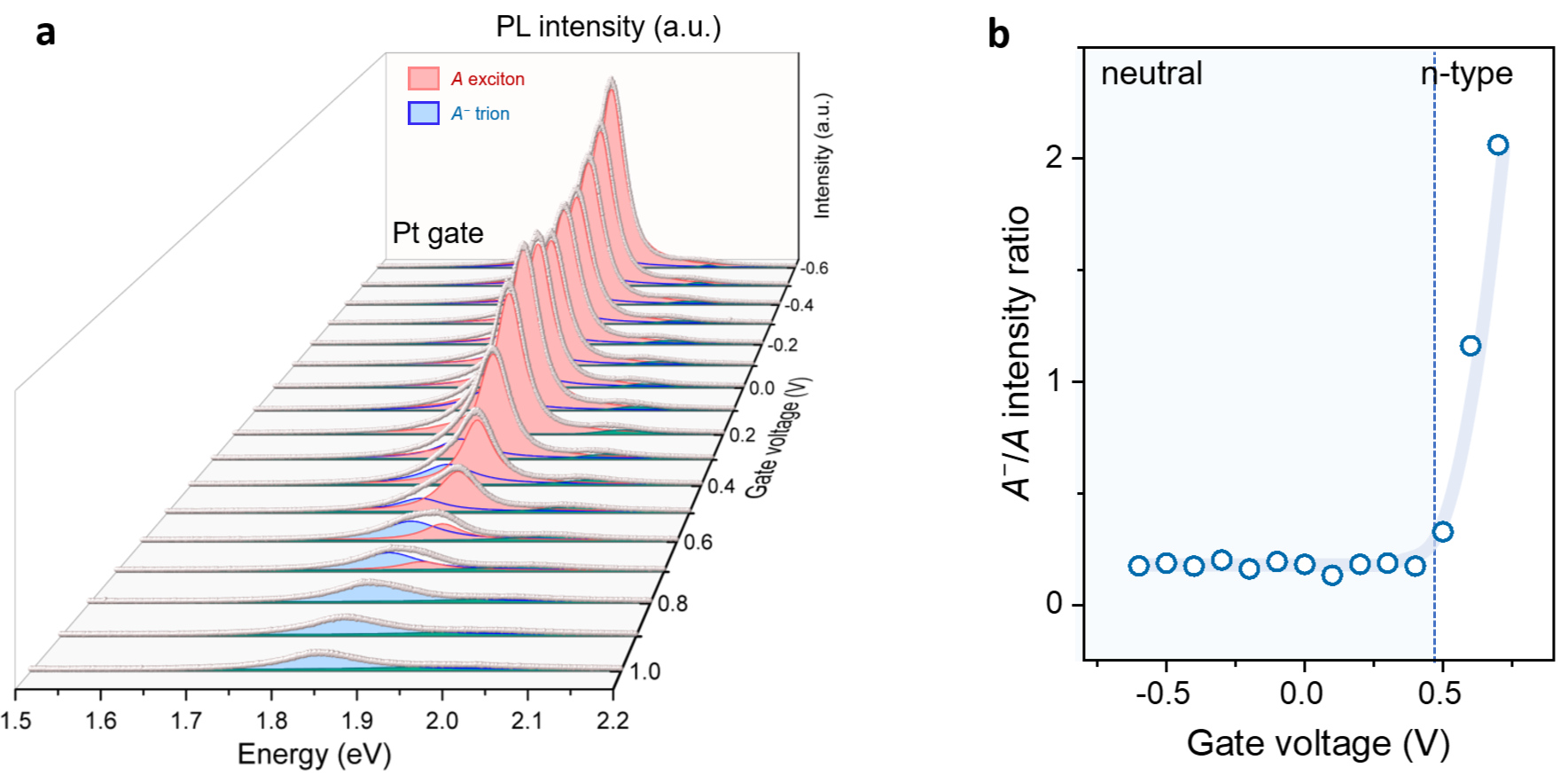


**Figure S7** Gate-dependent PL spectra of monolayer MoS_2_ on ZrO_2_ with Pt gate. (a) Deconvoluted PL spectra under varying gate voltages, showing the evolution of exciton and trion features. (b) The extracted trion-to-exciton intensity ratio as a function of gate voltage, revealing a transition from neutral to n-type doping around +0.5 V. The MoS_2_ remains in a nearly electron-neutral state under zero gate bias, indicating minimal electron doping on the ZrO_2_ dielectric with Pt gate.


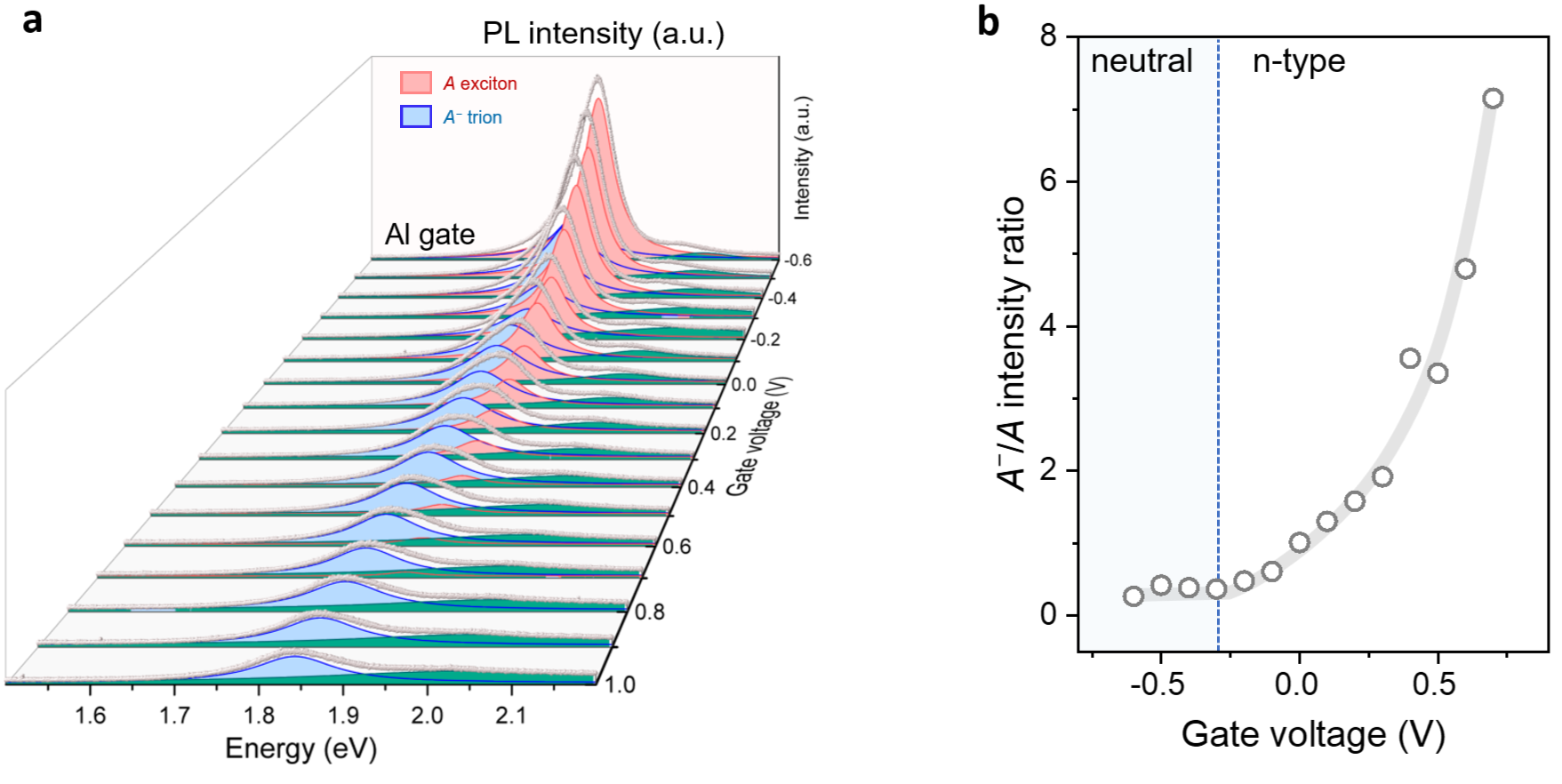


**Figure S8** Gate-dependent PL spectra of monolayer MoS_2_ on ZrO_2_ with Al gate. (a) Deconvoluted PL spectra under varying gate voltages, showing the evolution of exciton and trion features. (b) The extracted trion-to-exciton intensity ratio as a function of gate voltage, revealing a transition from neutral to n-type doping around -0.3 V. The MoS_2_ exhibits a trion-dominated (n-type) emission at zero gate bias, indicating notable electron accumulation induced by the low work function Al gate.

Using 6 nm ZrO_2_ as the dielectric, the critical transition voltage difference between with devices with Pt gate and Al gate is 0.8 V. The corresponding change in electron concentration can be estimated from the electrostatic relation:

$$\Delta n= C\cdot\Delta V=\frac{\varepsilon_{\mathrm{ZrO}_{2}}\varepsilon_{0}}{\tau_{\mathrm{ZrO}_{2}}}\cdot\Delta V$$

Substituting the experimental parameters yields *Δn* ~ 1.7×10^13^ cm^−2^_,_ confirming that gate work function engineering enables substantial carrier density modulation when paired with ZrO_2_.


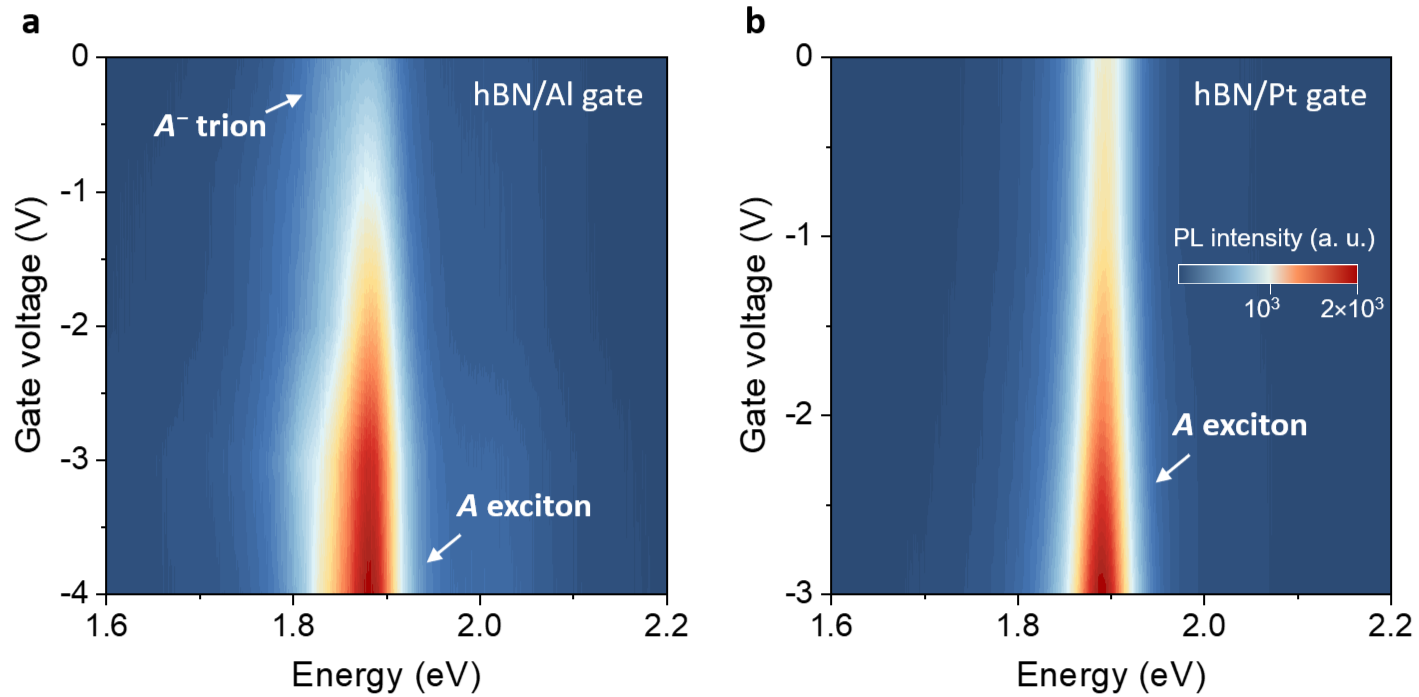


**Figure S9** Gate-dependent PL mapping of monolayer MoS_2_ on hBN with different gate metals. (a) MoS_2_ on hBN with Al gate exhibits trion-dominant emission at zero gate bias, indicating strong electron doping induced by the low work function metal. (b) In contrast, the sample with a Pt gate shows exciton-dominant features at zero bias, consistent with a near-neutral state. These observations are identical to the trends observed in ZrO_2_-based devices.

The change in critical voltage is approximately 2 V, leading to the *Δn* ~ 4.3×10^12^ cm ^−2^ for devices on hBN.


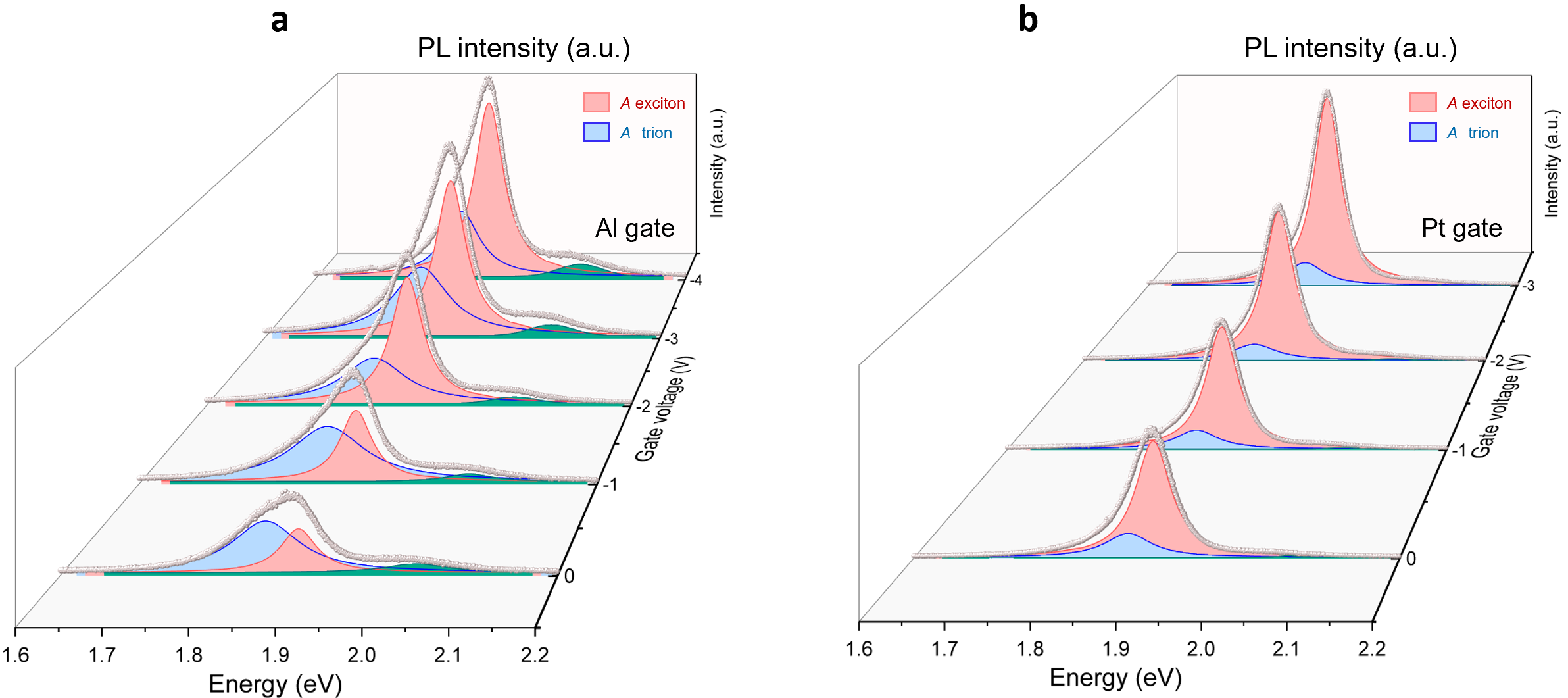


**Figure S10** Deconvoluted PL spectra of hBN-based devices with (a) Al gate and (b) Pt gate under varying gate voltages. At zero gate bias, the Al-gated device exhibits a trion-dominant emission, whereas the Pt-gated device shows an exciton-dominant peak.


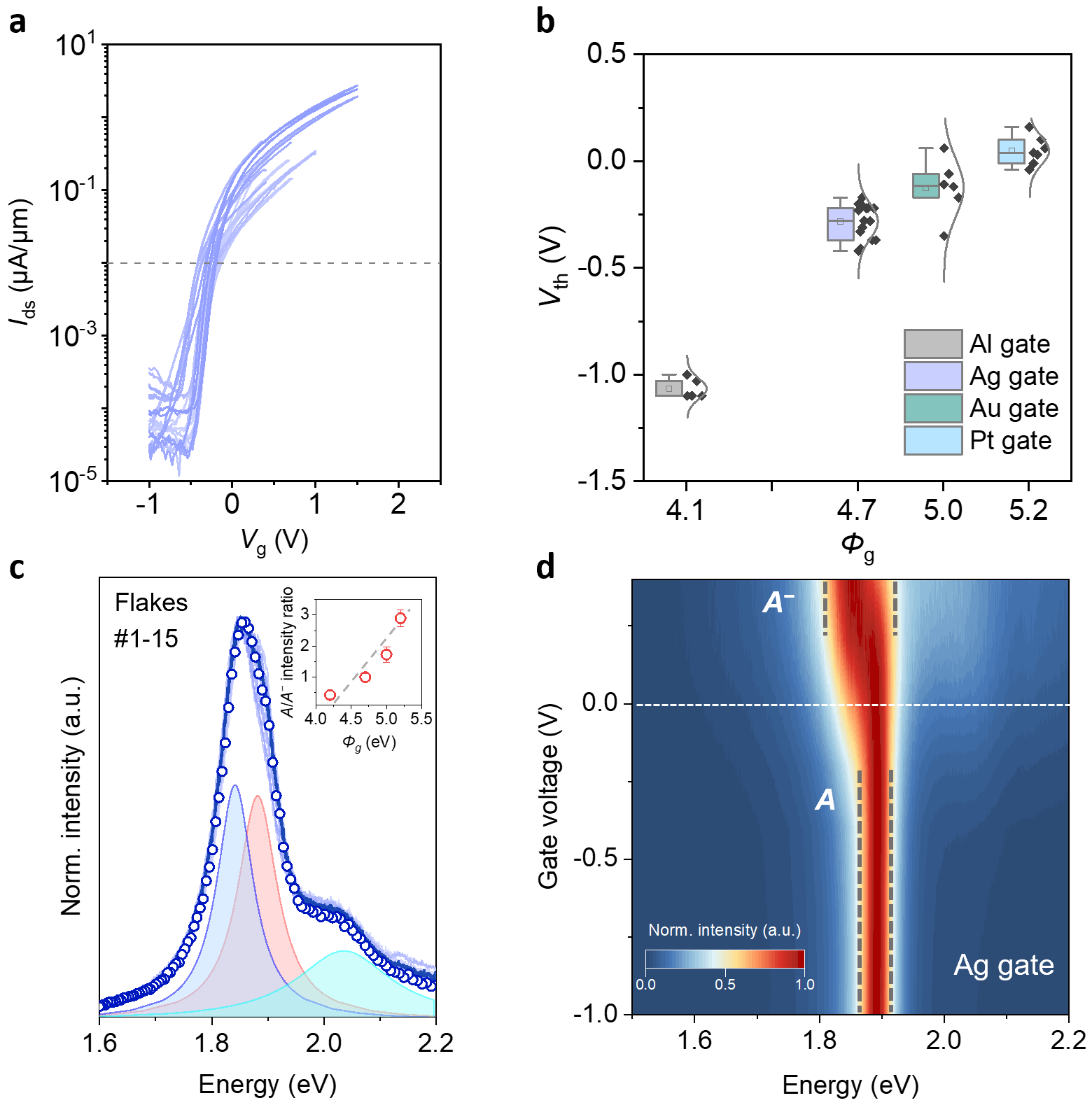


**Figure S11** Electrical and optical properties of Ag-gated monolayer MoS_2_ FETs. (a) Transfer characteristics of 15 representative devices. (b) Extracted V_th_ from panel (a), plotted as a function of gate metal work function. (c) PL spectra collected from 15 different monolayer MoS_2_ flakes. Insets: Extracted exciton-to-trion intensity ratio as a function of gate metal work function. (d) 2D colour map of the gate-dependent PL intensity, indicating that at zero gate bias the device is near the exciton-trion transition regime, where comparable contributions from both species are observed.


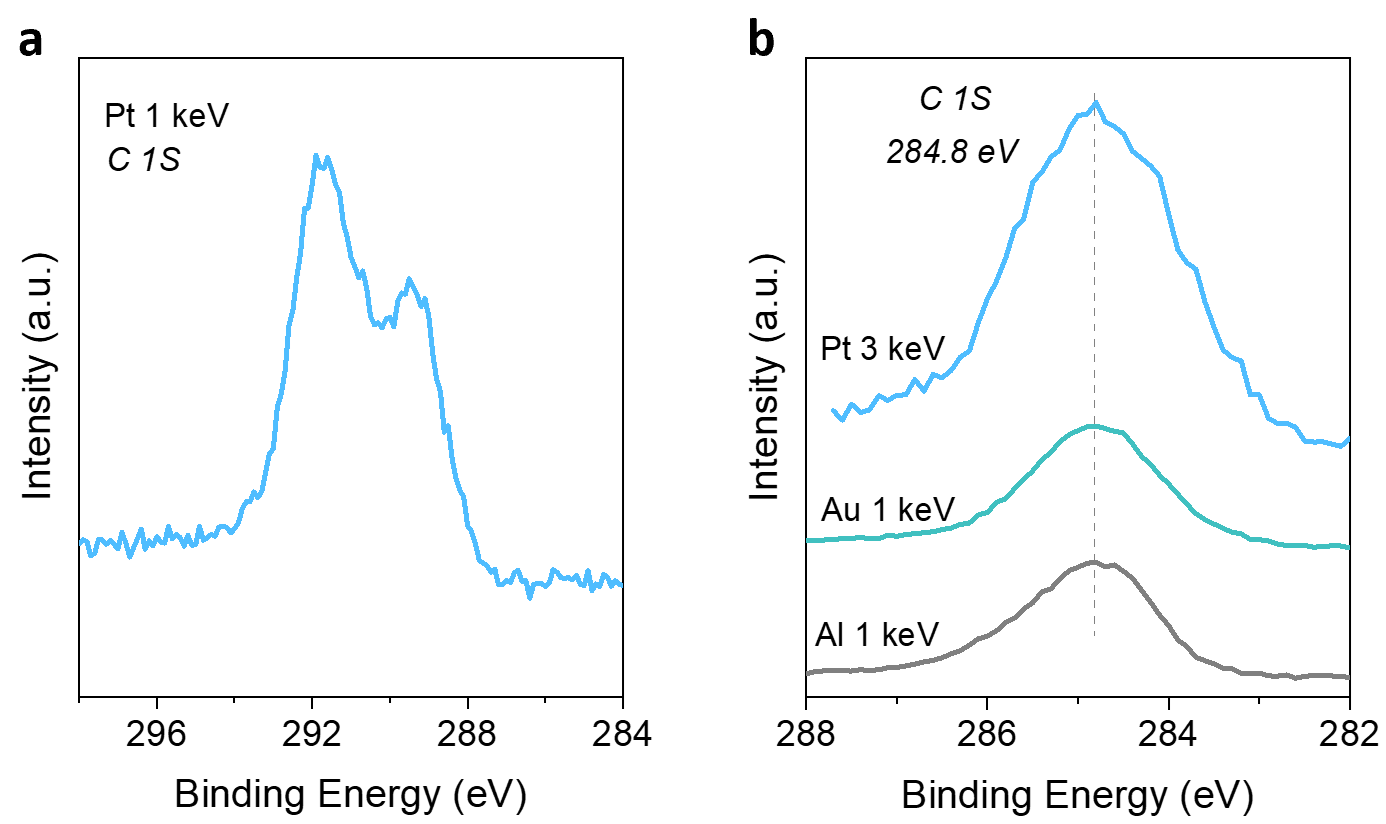


**Figure S12** Synchrotron XPS analysis of carbon peaks for calibration. (a) High-resolution C 1*s* XPS spectrum of MoS_2_ on hBN with Pt gate, acquired using 1 keV excitation energy. The observed peak splitting likely originates from physiosorbed surface contamination. Therefore, for this sample, the X-ray energy was increased to 3 keV to ensure reliable calibration of the Mo 3*d* core level. (b) Calibrated C 1*s* peak positions for samples with different metal gates for all measurements.


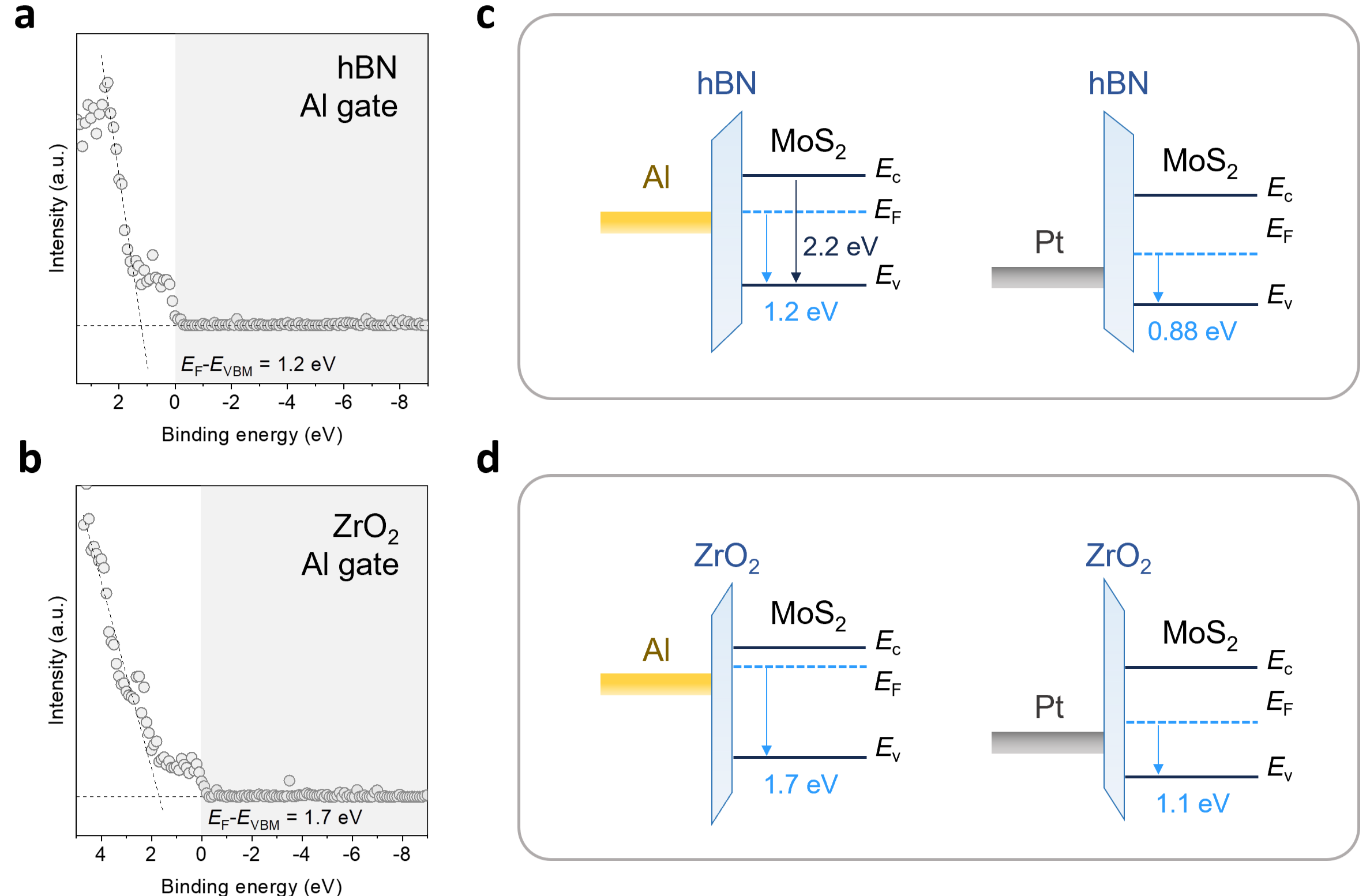


**Figure S13** Valence band (VB) spectra of devices with Al gate and (a) hBN and (b) ZrO_2_ as dielectrics, with corresponding band diagrams shown in (c) and (d).

Based on the band alignment, the carrier concentration in monolayer MoS_2_ with different metal gates and dielectrics can be calculated through the following formula suitable for 2D electron systems:

$n=N_{c}ln[1+exp(\frac{E_{F-}E_{i}}{kT})]$,

where $N_{c}=g_{v}g_{s}2\pi m_{e}^{*}kT/h^{2}$, where $g_{v}$and $g_{s}$being the valley and spin degeneracies, $m_{e}^{*}$ is the effective electron mass (0.45-0.73$m_{0}$), *k* is Boltzmann’s constant, T is estimated at 300 K, and *h* is Planck’s constant. This gives $N_{c}$ on the order of 10^12^ cm^−2^.

By substituting E_F_−E_i_ = 0.1 eV and 0.6 eV, the extracted electron concentrations are $n_{hBN}^{Al}=$ 3.9×10^12^ cm^−2^, and $n_{\mathrm{ZrO}_{2}}^{Al}=$ 2.3×10^13^ cm^−2^. For the devices with Pt gates, the Fermi level is located near mid-gap, yielding approximately intrinsic carrier concentration.

**Table S1** Electron concentration difference (*n*_Al-Pt_) between Al-gated and Pt-gated devices using hBN and ZrO_2_ dielectrics. The values are obtained from FET electrical measurements, gate-dependent PL, and VB measurement using synchrotron XPS. All electron concentrations are reported in units of cm^−2^.


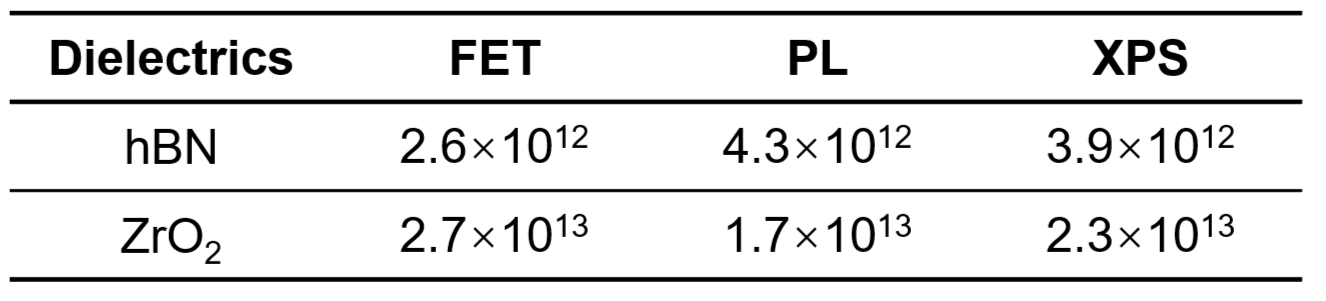


For the FET measurements, the change in V_th_ is extracted and substituted into the electrostatic relation

$$\Delta n= C\cdot\Delta V_{th}/q=\frac{\varepsilon_{dielectric}\varepsilon_{0}}{\tau_{\mathrm{dielectric}}}\cdot\frac{\Delta V_{th}}{q}$$

Where$\varepsilon_{dielectric}$and $\varepsilon_{0}$ are the dielectric constant and vacuum permittivity, respectively; $\tau_{\mathrm{dielectric}}$ is the dielectric thickness; $\Delta V_{th}$ is the threshold-voltage difference between Al- and Pt-gated FETs; and *q* is the elementary charge.

The procedure used to calculate the carrier concentration difference from gate-dependent PL and synchrotron XPS are described in Figures S6, S7, and S10.

The consistent values demonstrate the effectiveness of gate work function engineering in tuning the electron concentration in hBN and ZrO_2_-based devices.


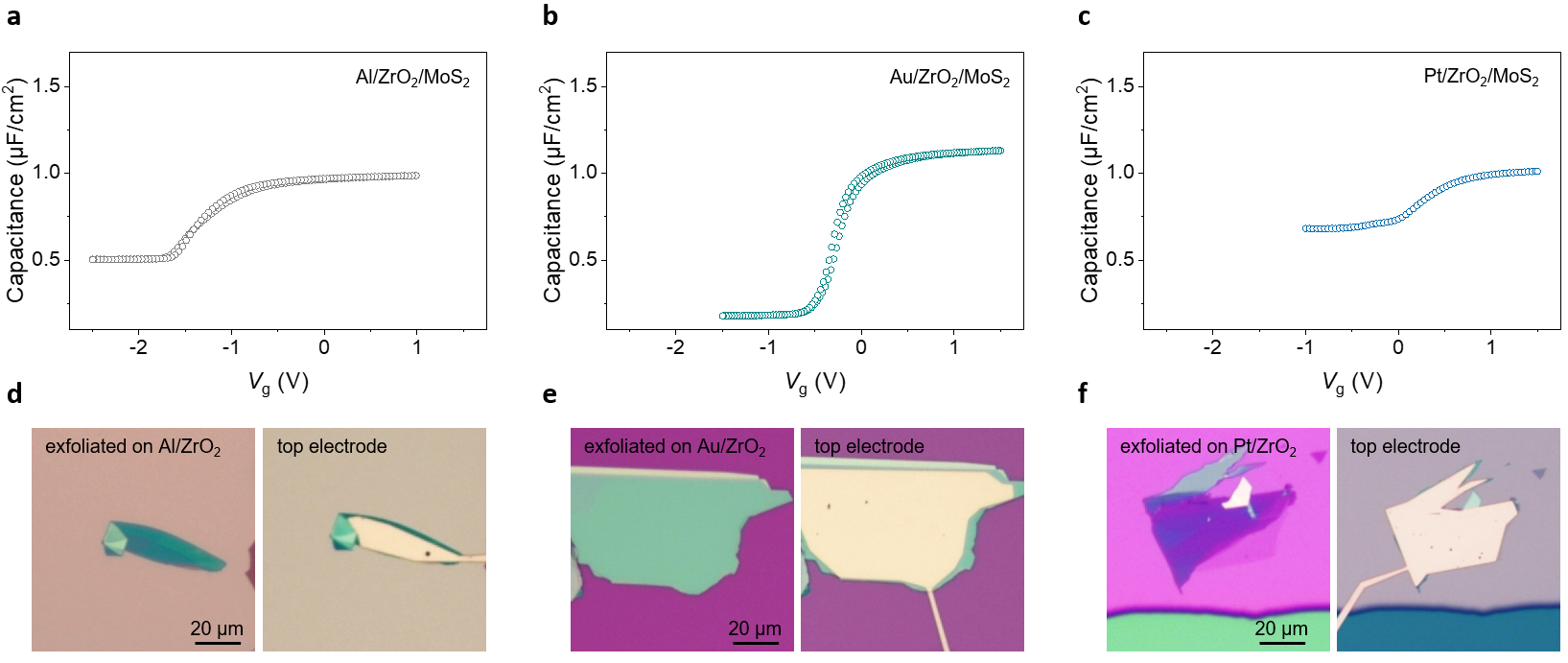


**Figure S14** Capacitance -voltage (C-V) characteristics (per unit area) of metal/ZrO_2_/MoS_2_ MOSCAPs with (a) Al, (b) Au, and (c) Pt gates, and corresponding OM images of the devices (d-f).

The variation in initial capacitance arises from differences in MoS_2_ thicknesses, which is inherently random during exfoliation. For the Al-gated device ($\tau_{\mathrm{MoS}_{2}}$=10 nm), the capacitance of MoS_2_ is calculated by $C_{\mathrm{MoS}_{2}}=\frac{\boldsymbol{\varepsilon}_{0}\boldsymbol{\varepsilon}_{r}}{\tau_{\mathrm{MoS}_{2}}}$. The corresponding system capacitance is estimated by $\frac{1}{C_{\mathrm{initial}}}=\frac{1}{C_{\mathrm{MoS}_{2}}}+\frac{1}{C_{\mathrm{ZrO}_{2}}}$, giving a value of $C_{\mathrm{initial}}=0.424$ μF/cm^2^. For the Au-gated device ($\tau_{\mathrm{MoS}_{2}}$=40 nm), the larger thickness reduces $C_{\mathrm{initial}}$ to 0.14 μF/cm^2^. For the Pt-gated device ($\tau_{\mathrm{MoS}_{2}}$=5 nm), $C_{\mathrm{initial}}=0.61$ μF/cm^2^. The small bulge in the spectrum reflects flake thickness non-uniformity. All calculated values are in good agreement with experimental results.


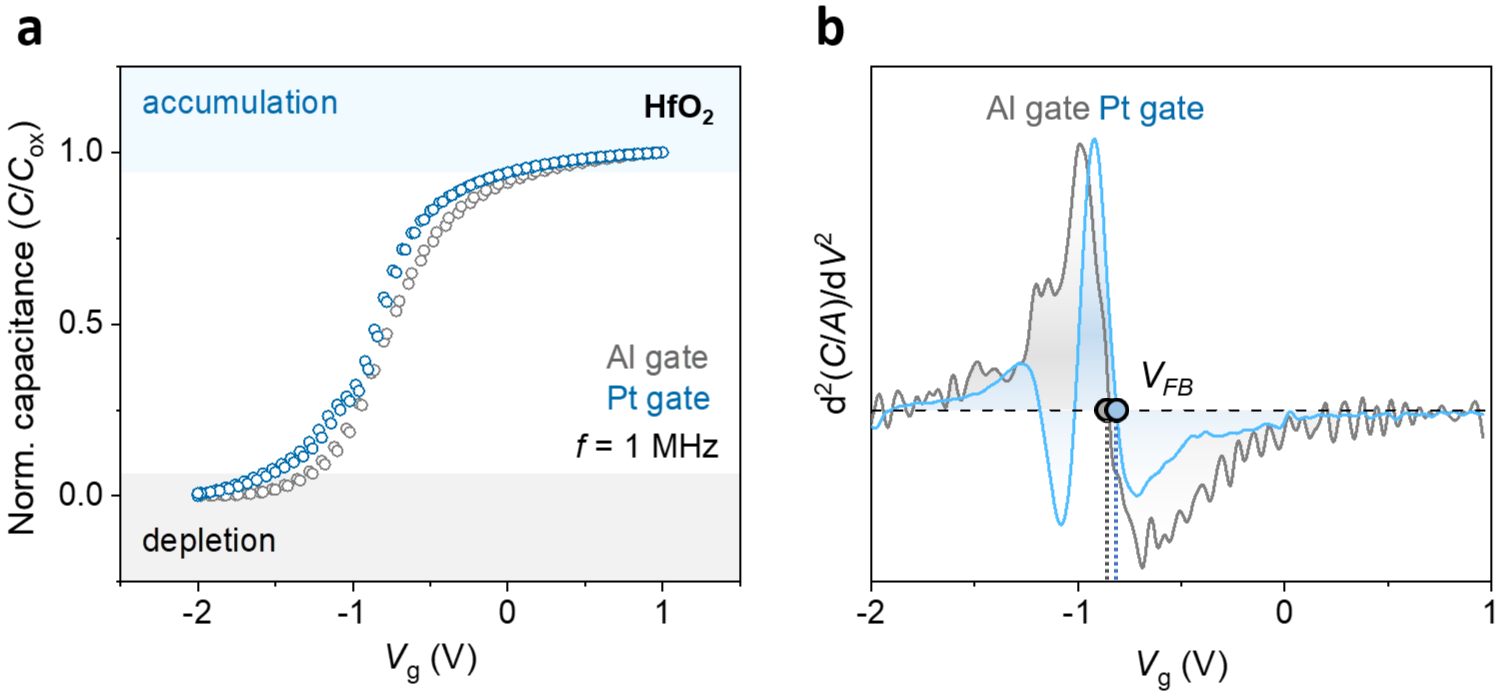


**Figure S15** (a) Normalized capacitance-voltage (C-V) characteristics of HfO_2_-based MOSCAPs with different bottom metal gates. (b) Corresponding second-derivative curves used to extract the flat-band voltage (V_FB_). The devices exhibit nearly identical C-V characters, and the V_FB_ remains pinned at approximately −0.8 V, independent of the gate work function.


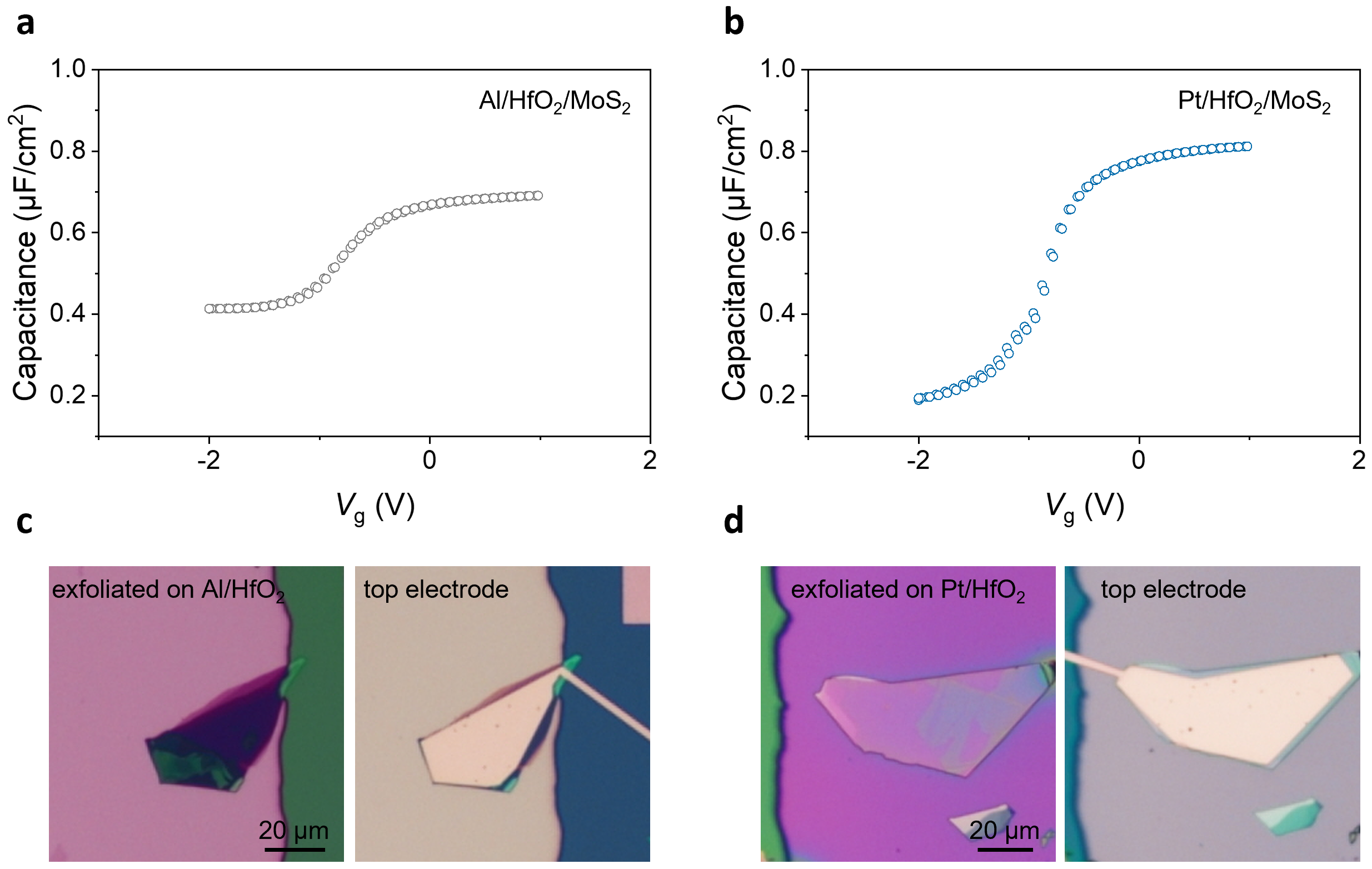


**Figure S16** Capacitance per unit area-voltage (C-V) characteristics of metal/ZrO_2_/MoS_2_ MOSCAPs with (a) Al and (b) Pt gate, and corresponding OM images of the devices (c-d).

For the Al-gated device, $\tau_{\mathrm{MoS}_{2}}$=10 nm, leading to the $C_{\mathrm{initial}}=0.37$ μF/cm^2^. For the Pt-gated device, $\tau_{\mathrm{MoS}_{2}}$=40 nm, leading to smaller $C_{\mathrm{initial}}=0.14$ μF/cm^2^. These calculated values are also consistent with the measured results.


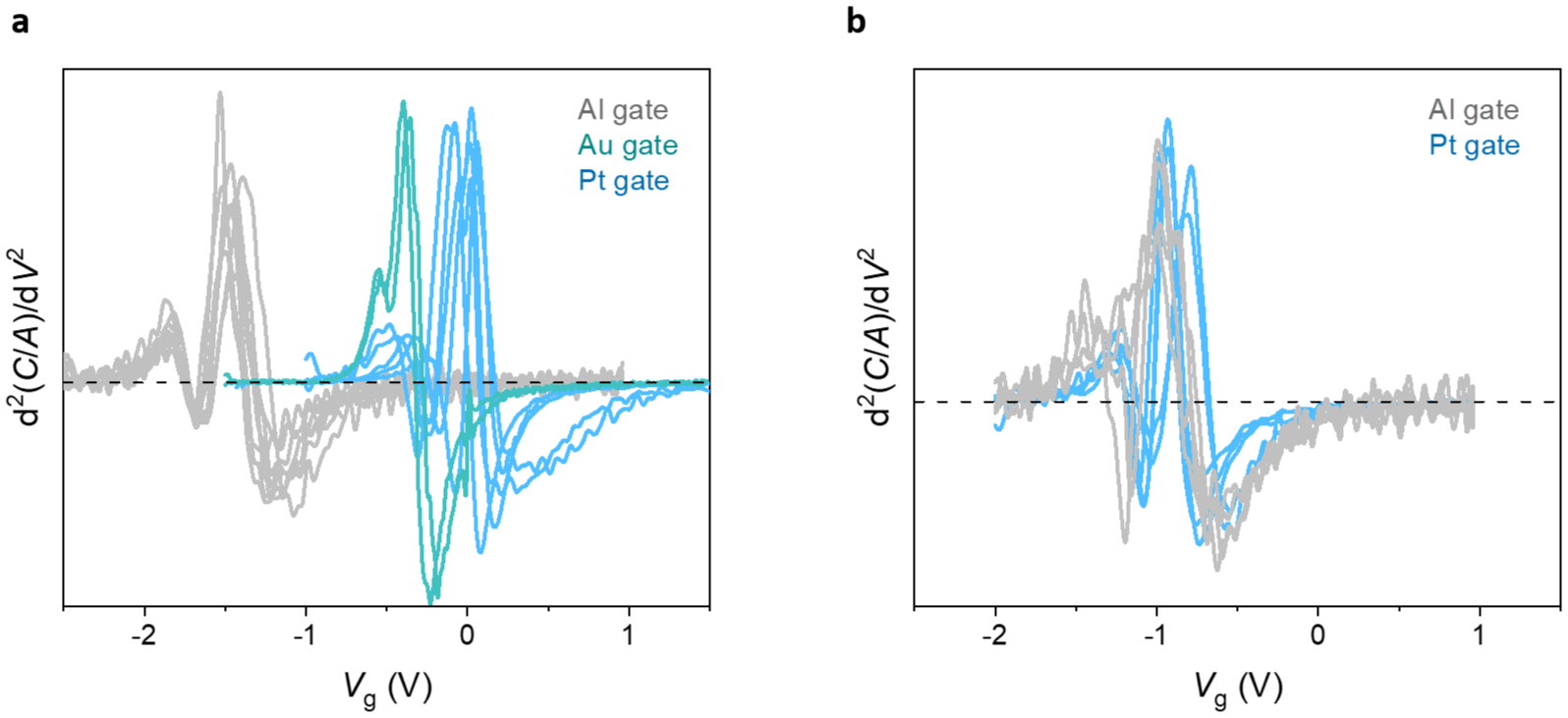


**Figure S17** Second derivative of the C-V curves from multiple devices (a) with ZrO_2_, and (b) with HfO_2_ as the dielectric to extract the flat band voltage. The shifts between different metal gates are significant in the devices with ZrO_2_. However, the curves with different metal gates almost completely overlap in the devices with HfO_2_.


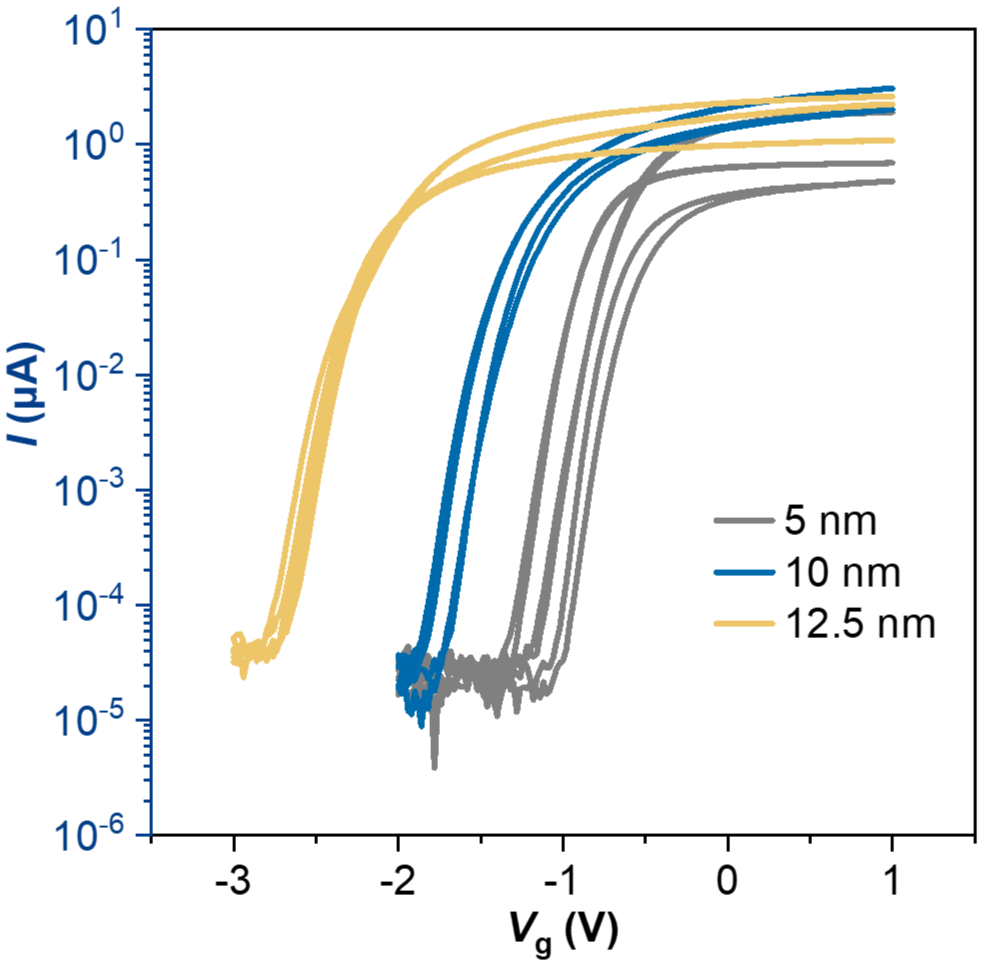


**Figure S18** Transfer curves of monolayer MoS_2_ FETs based on Au gate and HfO_2_ dielectrics of varying thickness. Devices with thicker HfO_2_ exhibit progressively more negative V_th_, indicating strong EOT-dependent V_th_ shifts arising from interfacial and oxide charge trapping.


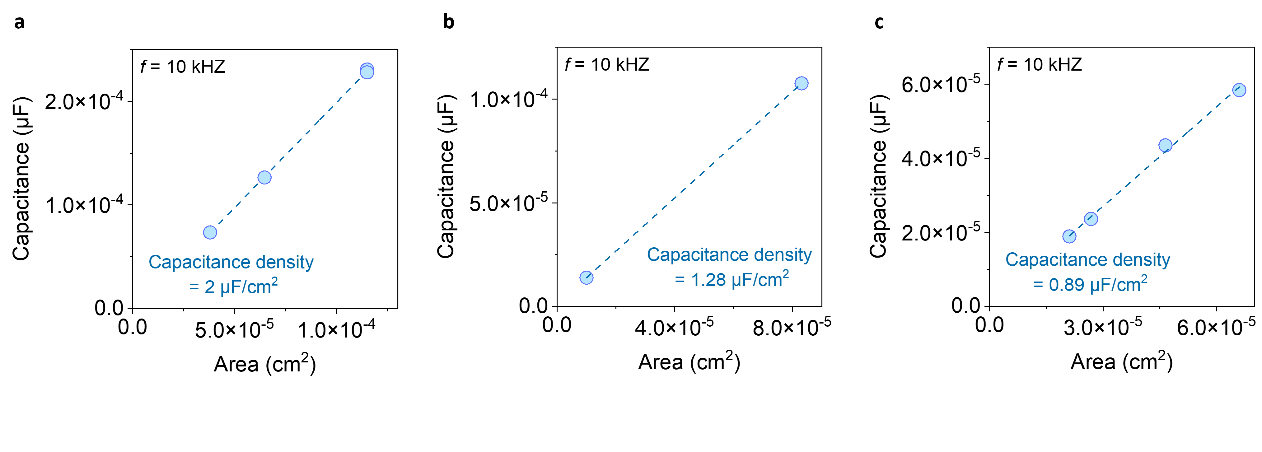


**Figure S19** Extracted capacitance of the MIM structures for ZrO_2_ with thicknesses of 6.5 nm (a), 12 nm (b) and 20 nm (c). The corresponding extracted dielectric constants are 14.3, 17.3, and 20.1, respectively. The discrepancy between these values may arise from a non-ideal interfacial layer between the metal and the dielectric.
